# Regenerative Medicine in South Korea: Bridging the Gap Between Authorization and Reimbursement

**DOI:** 10.3389/fbioe.2021.737504

**Published:** 2021-08-30

**Authors:** Dong-Sook Kim, Geunwoo Lee, Hyungyung Cho, SeungJin Bae

**Affiliations:** ^1^ Department of Research, Health Insurance Review and Assessment Service, Chuncheon, South Korea; ^2^ College of Pharmacy, Ewha Womans University, Seoul, South Korea

**Keywords:** regenerative medicine, marketing authorization, pricing, reimbursement, conditional coverage

## Abstract

Regenerative medicine (RM) has considerable potential to address the needs of aging-related and uncurable diseases. However, its incorporation into reimbursement of health insurance benefits poses many challenges, including uncertain evidence and insufficient investment. This paper examines the wide gap between manufacturers, regulatory bodies, and health technology bodies regarding reimbursements for RMs focused cell therapy products. In this mixed-methods study, we first analyzed the sales of RMs approved in South Korea. In addition to exploring beliefs related to the market value of RMs, in-depth interviews were conducted with 24 experts (17 from bio-industries, two from the regulatory body, three from a health technology assessment (HTA) body, and two from the Pharmaceutical Benefit Coverage Assessment Committee [PBCAC]). Lastly, we surveyed PBCAC members about the market value of RMs. In total, 15 of the 20 developed cell therapy products are on the market in South Korea, and amounted to 0.24% of total pharmaceutical expenditures in 2018. We identified a wide gap between stakeholders and regulators regarding the market value and pricing of RMs. The interviewees from the pharmaceutical manufacturer association raised the issue of rising manufacturing costs and proposed a specific pricing policy for RMs. To bridge the gap between approval and reimbursement, stakeholders demand an alternative framework of value-based pricing. Conditional health insurance reimbursement may be an alternative to the traditional process in order to generate evidence of the effects of RMs using “risk-based” or “outcome-based” approaches.

## Introduction

As medical technology advances, more diseases than ever can be prevented and treated, but there are still many diseases that cannot yet be cured. Stem cells and progenitor cells, which have the ability to generate or regenerate functional cells and tissues, have a tremendous potential to bring about changes in the medical field (Edgar et al., 2013).

Regenerative medicine (RM) uses stem cells and progenitor cells to repair or reconstruct damaged functional cells and tissues. RMs have the potential for market growth and could change the medical field (Edgar et al., 2013), and are thus considered a promising approach for curing diseases that are difficult to remedy using traditional treatments (Buzhor et al., 2014). RMs involve the mechanisms of proliferation and differentiation, paracrine effects, and migration and apoptosis (Fu et al., 2019), which allow cell therapies to regenerate damaged cells or tissues and stimulate the process of endogenous tissue self-healing by the nutrient effects mediated by cytokines and the secretion of growth factors to regenerate damaged cells or tissues (Buzhor et al., 2014). This treatment cures the fundamental cause of diseases (Chhabra and Brayman, 2013; Hinrichs and Rosenberg, 2014), thus making it a new treatment paradigm to meet currently unmet medical needs. Given the potential uses of RM, its market is growing very rapidly. The annual and cumulative compound annual growth rate (CAGR) in financing for the stem cell therapy sector were 31.5 and 44.8% from 1999 to 2016, respectively. This is a very significant growth rate compared to the 26.4% CAGR for the overall healthcare market (Ng et al., 2017).

In Korea, RMs is defined as “cell therapy products are medicines manufactured by manipulating living cells of humans or animals by physical, chemical, or biological methods, such as culturing, propagation, or selection *in vitro*” by regulation of RM approval since 2008 (MFDS, 2008) and Good Manufacturing Practice (GMP) guideline of RMs since 2010 (MFDS, 2010) under the Pharmaceutical Affairs Act. These regulation and guidelines requires facilities standard and validation process of RM manufacturing. In 2020, to encourage the approval and management of biopharmaceuticals, the Act on Advanced Regenerative Medicine and Advanced Biopharmaceuticals (ARMAB) was enacted (Ministry of Health and Welfare, 2020). According to this act, advanced RMs has been expanded as “the use of human cells to regenerate, restore, or form a human body structure or function, or to treat or prevent disease. Among biopharmaceuticals, there are cell therapy products, gene therapy products, tissue engineering products, and advanced bio-convergence products”. To encourage the fast approval of RMs and biopharmaceuticals, the ARMAB contains conditional approval or expedited review in case where there is no alternative treatment and treatment for serious or rare disease.

However, RMs have a very high manufacturer price (USD 110,920–814,780) (Seoane-Vazquez et al., 2019). The use of living cells in RMs is associated with difficulties in the manufacture, transport, and delivery of therapies (Department for Business, Innovation and Skills, 2011), which are the key drivers that raise the manufacturing cost of RMs. Cell growth media, which are used to cultivate and maintain cells, account for 36% of the manufacturing cost, and a cold chain for cell therapy products needs to be maintained from the process of harvesting cells until the administration of treatment, even during the processing and storage steps (Lipsitz et al., 2017). Furthermore, to prevent microbial contamination and maintain specific properties of the living cell, the overall manufacturing process of living cells needs sterilization and aseptic testing (Giancola et al., 2012). Furthermore, since one of the main characteristics of RM is that it involves personalized treatment, individualized cell therapy must be adjusted according to patient-specific profiles since person-to-person and population differences exist (Arjmand et al., 2017). Therefore, RMs do not have the economic benefit of mass production for a large number of patients. Patient-specific therapies entail a linear increase in associated manufacturing costs (Lopes et al., 2018). Not surprisingly, because of high costs and uncertainty regarding the cost-effectiveness of cell therapies, deciding the appropriate reimbursement for cell therapies remains a challenge (Shukla et al., 2019).

This study analyzed the market access status of RMs focused cell therapy products in South Korea and summarized the challenges and improvement measures related to reimbursement of health insurance benefit listings. First, we analyzed the sale of RMs approved in South Korea. Second, to examine beliefs regarding the market value of RMs, in-depth interviews were conducted with 24 experts. Lastly, we conducted a survey about how to evaluate the market value of RMs with members of the Pharmaceutical Benefit Coverage Assessment Committee (PBCAC).

## Methods

### Review on Policy of RM Reimbursement

We searched the regulations of the Korean Ministry of Food and Drug Safety (MFDS) and special legislation affecting regenerative medicine in Korea (MFDS, 2020). We also reviewed the Korean National Health Insurance (NHI) benefit package (Gong et al., 2020).

### Analysis of Utilization of RMs

A total of 20 RMs were approved as of September 2020, and five of them had been withdrawn from the market. Four RMs are reimbursed by health insurance. We used NHI claims data from the Health Insurance Review and Assessment Service (HIRA), which covers almost 98% of the total population of Korea. NHI claims data consist of patients’ general information, diagnosis, healthcare service utilization, and all medication use. We also used HIRA supply data pertaining to reimbursed and non-reimbursed medicines from pharmaceutical wholesalers to medical institutions and pharmacies (Kim et al., 2017). We extracted annual data on the sales volumes of all RMs from 2011 to 2018. We classified the therapeutic class according to the Anatomical Therapeutic Chemical (ATC) classification system outlined by the World Health Organization Collaborating Centre (WHOCC, 2020).

### Group Interview

In-depth interviews were conducted in three groups: stakeholders (bio-pharmaceutical industry executives), regulators (MFDS and HIRA officials), and PBCAC members. We conducted interviews with 17 people from 10 bio-industrial companies, two MFDS officials, three HIRA officials, and two experts from the Economic Evaluation Subcommittee of PBCAC, for a total of 24 interviews. The interviews were conducted in groups across a total of seven sessions from March 21, 2016, to April 28, 2016. The interviews proceeded for approximately 90 min each and researchers distributed questionnaires about the main topic. The interviews were conducted to collect feedback on the need for preferential pharmaceutical pricing for cell therapies, screening for preferential targets, pricing evaluation criteria that reflect the specificity of RM treatments, and post-management payment plans. Although the focus of the interviews differed according to the group of people being interviewed, they generally centered on the following topics: the market value of RMs, criteria for the detailed assessment of RMs, and post-management payment plans for costs incurred by RM treatment.

To address the topic of the value of cell therapy products, the researchers prepared research materials in advance that included cell therapy approval reviews, reimbursement status data, and cell therapy characteristics. We presented our prepared materials before soliciting the opinions of group representatives. Beliefs and attitudes regarding the importance of considering the value and innovativeness of RMs for determining the degree of reimbursement were collected from each stakeholder. To collect their beliefs and attitudes regarding the importance of creating detailed evaluation criteria to account for the special circumstances of cell therapies, we asked the interviewees to share their opinions freely on how best to change the drug price system in the future and to share their experiences related to this topic. The MFDS officials were asked where improvements were needed in the approval process of cell therapies and their future plans with regard to the approval process. Lastly, discussions on follow-up management measures if preferential prices were implemented were also conducted.

The interviews were digitally recorded and transcribed. The data were thematically analyzed and systematically coded using the framework approach (Pope and Mays, 2013). Initially, two members of the research team independently coded and cross-checked the data. Once themes and codes had been determined, the final stage of analysis involved checking and discussing the data interpretation.

### Survey

A questionnaire was distributed to 66 members of PBCAC from April 24, 2017, to April 28, 2017, with a total of 17 members responding. The survey included questions about their beliefs related to the value of cell therapies, how to determine the cost of cell therapies, and hypothetical scenarios for cost-effectiveness.

## Results

### Market Authorization, Pricing, and Reimbursement of RMs in South Korea

#### Approval System of RMs

**FIGURE 1:**
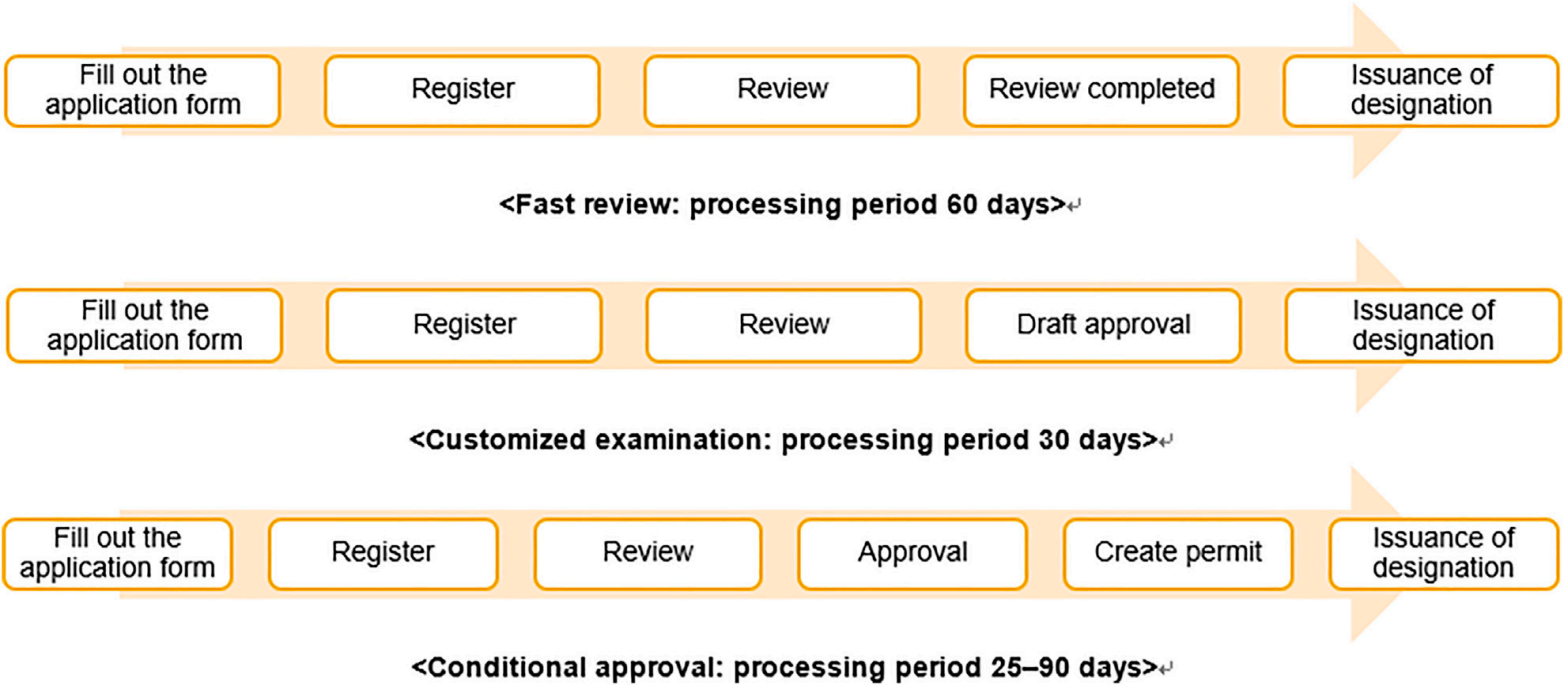
Procedures and timeline for rapid processing of RMs. Note: The processing procedures for designating advanced biopharmaceuticals as subject to rapid processing, for customized screening of advanced bio-medicine, and for conditional manufacturing and sales item approval.

South Korea passed the Act on Advanced Regenerative Medicine and Advanced Biopharmaceuticals in August 2019 and implemented it in August 2020. The purpose of this act was to contribute to the improvement of public health by preparing a system for the safe management, support, and commercialization of RM, expanding patient treatment opportunities, and strengthening safety management by enabling clinical research related to cell therapies and supporting a rapid approval process (Figure 1).

According to Article 36 of the act, the MFDS may place applications for applied cell therapies under expedited review under certain conditions, such as 1) if there is no alternative treatment and the purpose is to treat serious and life-threatening diseases, such as cancer, 2) if the purpose is to treat rare diseases under the Rare Disease Control Act, and 3) if the purpose is to prevent or treat a pandemic of infectious diseases, such as ones resulting from acts of bioterrorism, and other infectious diseases under the Act on the Prevention and Management of Infectious Diseases.

Cell therapies approved to undergo the expedited review system are handled as follows (Figure 1). Before applying for the drug approval, if a manufacturer can submit individual data for each development process and request it to be reviewed in advance, their application will be reviewed as a “customized examination.” If a manufacturer applies for item authorization on the basis of clinical trial data showing that the treatment works for surrogate outcomes that reasonably predict clinical benefits from the perspective of pharmacokinetics, pharmacology, pathophysiology, and similar fronts, a conditional approval may be granted on the condition of post-marketing safety management. Based on this new law, medical institution can conduct treatment of RM if clinical research is approved.

#### Reimbursement and the Decision-Making Process for Determining Pricing of RM in South Korea

South Korea has required positive listings according to value-based pricing since 2007. In general, if a drug’s price is higher than its alternative, economic evaluation data for the drug are required to be listed in the NHI benefit package (Gong et al., 2020). Once pharmaceutical companies submit a dossier for applying for reimbursement to the HIRA, the HIRA and Economic Evaluation Subcommittee review the cost-effectiveness data. Based on the results of this review, the PBCAC makes the final decision on reimbursement and pricing through negotiations between companies and the National Health Insurance Service.

Since 2017, the HIRA introduced exceptional criteria designed for products that “positively impact healthcare in general” (Cho et al., 2020), which may be applicable to RM. Currently, four cell therapies (Chondron®, Kaloderm®, Cupistem®, and Keraheal-Allo®) are formally approved in South Korea, and Cupistem® and Keraheal-alo® were listed based on their price being lower than alternative medicines (Cho et al., 2020).

### Sale of RMs According to ATC Classification

South Korea had the highest number of authorized RMs in the world between 2001 and 2010 (Shukla et al., 2019). In 2011, the world’s first stem cell therapy product, “Cellgram-AMI,” was approved in South Korea (Cho et al., 2020). A total of 20 RMs have been approved up to September 2020, and five of them were withdrawn from the market. A total of four RMs are eligible for reimbursement from health insurance providers (Supplementary Materials).

We used NHI claims data from the HIRA, which covers almost 98% of the total population in Korea. NHI claims data included patients’ general information, diagnosis, healthcare service utilization, and all medication use. In addition, we examined supply data pertaining to reimbursed and non-reimbursed medicines from a pharmaceutical wholesaler to medical institutions and pharmacies (Kim et al., 2017). We extracted yearly data on sales volumes of all RMs from 2011 to 2018. We classified the therapeutic class according to the ATC classification system outlined by the WHOCC (WHOCC, 2020).

In 2018, immunostimulants (ATC Code L03) accounted for the largest RM expenditures, followed by a blood product (ATC code B05). According to the analysis of NHI claims data and supply data for approved cell therapies, reimbursement-ineligible cancer treatments made up the largest expense, amounting to USD 24.52 million, and expenses related to mesenchymal stem cells used to treat cartilage in patients with degenerative osteoarthritis totaled USD 16.67 million.

**FIGURE 2:**
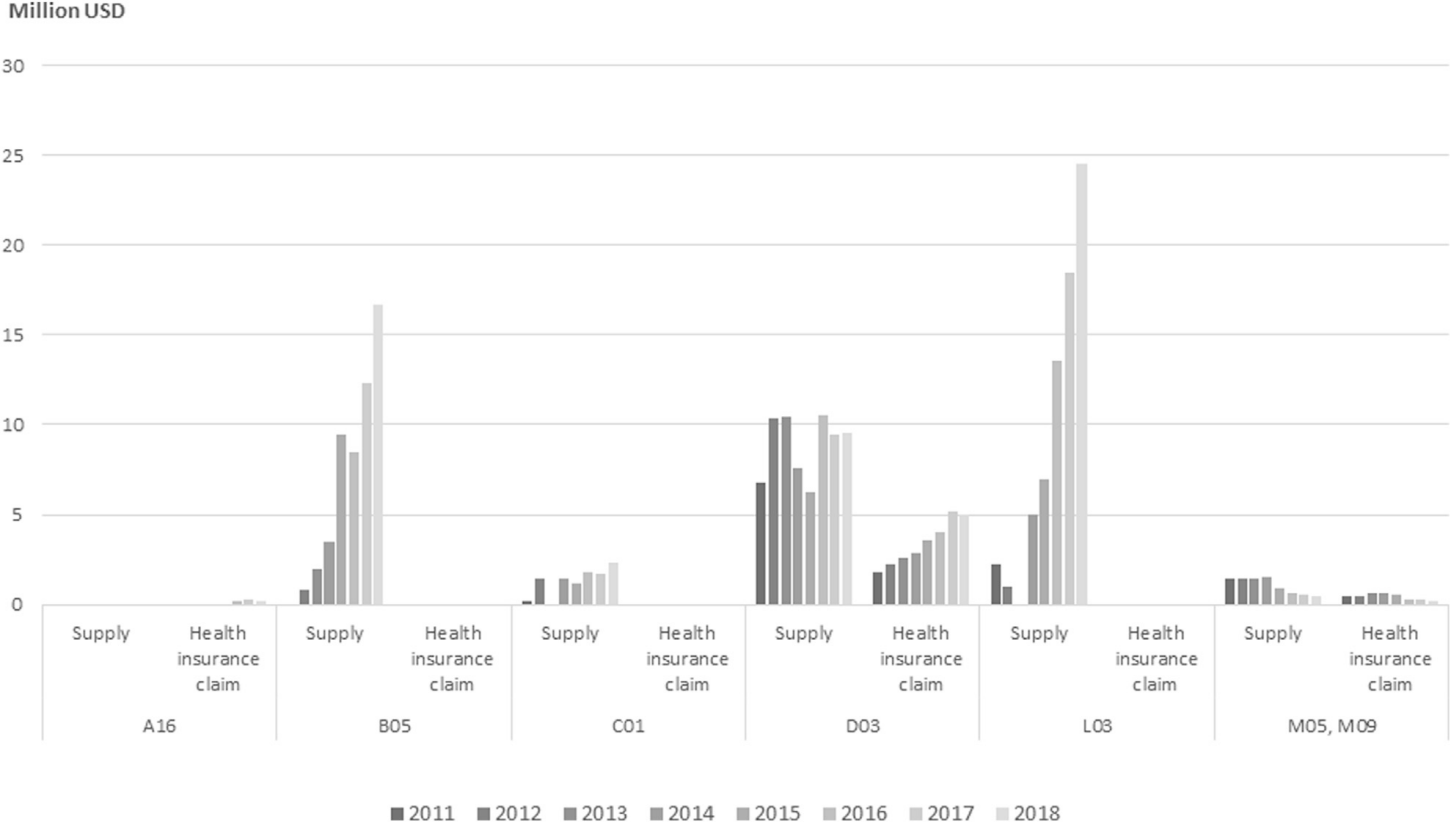
Annual expenditure of approved and reimbursed cell therapy products by ATC class from 2011 to 2018. Note: ATC, anatomical therapeutic chemical. A16 (Other alimentary tract and metabolism), B05 (Other blood products), C01 (Cardiac therapy), D03 (Preparations for treatment of wounds and ulcers), L03 (Other immunostimulants), M05, M09 (Musculo-skeletal system). No. of approved, No. of reimbursed drugs—A16 (1,0), B05 (2,0), C01 (1,0), D03 (7,2), L03 (2,0), M05 and M09 (2,1).

There are four RM treatments eligible for reimbursement, constituting only 0.08% of the 22,303 items on the 2018 list of treatments eligible for reimbursement. The cost of supplies used in RMs was USD 53.57 million, but claims for health insurance reimbursement amounted to USD 5.38 million, corresponding to only 0.04% of the total USD 14.89 billion in health insurance drug costs in 2018 and 0.24% of the USD 22.67 billion in drug costs supplied to medical institutions in 2018 (Figure 2).

### Interviews With Stakeholders (Manufacturers, Regulatory Body Officials, and HTA Officials)

Interviews were conducted in three groups: stakeholders (executives from biopharmaceutical associations and the traditional pharmaceutical industry), regulators (MFDS and HIRA officials), and the PBCAC. We collected opinions from 17 executives at 10 bio-industrial companies, two MFDS officials, three HIRA officials, and two experts from the Economic Evaluation Subcommittee of PBCAC (24 people in total) from March 21, 2016 to April 28, 2016. The interview topics were about the market value of RM, criteria for detailed assessment of RM, and post-management payment plans for costs incurred by RM treatment. Table 1 summarize their opinion.

**TABLE 1:** Expert Interview on value and pricing of RM.

	Bio industry	MFDS	HIRA	Economic evaluation subcommittee
**Challenge or Obstacle in RM Reimbursement**				
Valuation or innovation	RM can show clinical usefulness for intractable/rare/degenerative diseases	RM can distinguish material innovation based on the development phase		
**Pricing**				
Compensation	The drug price should be reflected in consideration of development costs	Manufacturing costs are not as high as other biomaterial medicines	Pricing rules are same	Considering risk-sharing system is needed
Economic assessment criteria	- Difficulty in submitting evidence that can be obtained at the time of authorization	- Clinical validity can be determined by the clinical trial structure	Recognizing the difficulty with cost-effectiveness evaluation	- Drug price assessment criteria and cost effectiveness assessment are separate issues
	- Pointing out a difficulty with cost-effectiveness assessment and providing economic proof			- Opposition to the other economic assessment standards for RM
	- Point out the need for evaluation criteria only for cell therapies			
**Future Improvement Task**				
Improve Market Access	Need to come up with a cost conservation plan during the drug price negotiation period			
Promote Market Use (uptake or option)		It should be encouraged to lay the level of evidence		It is needed to invest more scale

MFDS, Ministry of Food and Drug Safety; HIRA, Health Insurance Review and Assessment Service; PBCAC, Pharmaceutical Benefit Coverage Assessment Committee.

#### Bio-Industry

Bio-industry executives suggested that it was difficult to submit evidence of the safety and efficacy of RMs at the time of authorization to compare them with conventional new drugs intended for large numbers of patients. Furthermore, the safety and efficacy of drugs are assessed based on phase III clinical trials or conditional approval after small-scale phase II clinical trials in Korea. Even with conditional approval, a phase III clinical trial plan, a risk management plan, and measures to restrict use beyond the obtained permission limits should be submitted.

“It has expensive production costs due to the batches used to cultivate cells and difficulties in reducing labor costs since most of the tasks are labor-intensive. In the case of self-derived cell therapy, it is difficult to determine compensation after manufacture. Third, if an item is approved beyond the phase III clinical trial stage, it should also be discussed how that will be reflected in the drug prices.” (A1, A3, A5, A7, A9, A11, A13, A15, A16, A17)

“For rare and incurable diseases, RM may have effects that traditional treatments do not have, such as prolonging life, preventing the progress of diseases, and improving the quality of life. When effects that traditional treatments do not have are used as an effect indicator, it is difficult to assess the cost-effectiveness compared to conventional treatment.” (A2, A4, A5, A6)

“The cost-effectiveness assessment criteria of RM need to be compared against secondary effectiveness indicators.” (A1, A3, A5, A7, A14, A15)

#### Regulatory Body (MFDS)

MFDS officials noted the limitations of RM clinical trials. Cell therapies unavoidably result in some clinical uncertainty due to the small number of participants and less rigorous trial designs. In addition, officials said that, unlike cell therapies that require a great deal of manual work, other biologic drugs can be produced in large quantities at a much lower price using automation.

“For value, we would consider the potential for export and the specificity of the RM sector. Export and large-scale clinical trials are impossible for autologous-cell therapy, and there are manufacturing limitations, industrial limitations, and inherent limitations of patient cells.” (B1)

“As for the specificity of the RM, there is a slim chance of large-scale standardization, and this is why RM companies are relatively small compared to other biopharmaceutical companies. It can be considered that these problems can be solved through technology exports and global clinical trials. However, we did not think to consider the high manufacturing costs related to the specificity of RM treatments.” (B2)

#### HTA Body (HIRA)

HIRA members reported already knowing that it is difficult to determine the market value of RMs since the evidence level is lower than that for other new drugs. Further, they mentioned the need for a way to account for specific indicators of RMs, unlike conventional therapies.

“In Korea, we review an economic assessment using an incremental cost-effectiveness ratio, but it is difficult to evaluate the cost-effectiveness of RM derived from intermediate outcomes that have been proven to be relevant to the final assessment indicators.” (C1, C2, C3)

#### Experts From the Economic Evaluation Subcommittee of the PBCAC

Experts from the Economic Evaluation Subcommittee of the PBCAC argued that there was a need to assess the market value of cell therapies in a different way from the existing framework so that the industrial value of cell therapies is accounted for.

“The idea could be to utilize the risk-sharing system for the production of evidence. Risk-sharing systems can be utilized initially through several case reports of RMs that worked, and later, clinical indicators can determine management after the cell therapies are listed by the NHI.” (D1)

“I think that it is difficult to prove the economic value of RMs. Instead, they say that if there is no alternative drug, comparison through best supportive care or natural progression can be considered when selecting a new drug alternative. Even if there is an alternative drug, direct comparison is difficult, and indirect comparison is likely to be difficult for assessing cost-effectiveness due to heterogeneity among patients. In consideration of these points, we can follow the economic assessment exception system once the effectiveness and safety of cell therapies are confirmed, but I am opposed to revising existing economic assessment guidelines and principles.” (D2)

### Survey of the PBCAC

The survey was conducted by distributing a questionnaire to 66 members of the PBCAC from April 24, 2017 to April 28, 2017, and 17 members responded. The survey asked questions about respondents’ beliefs and opinions regarding the market value of cell therapies, how to evaluate the market value of cell therapies, and hypothetical scenarios for cost-effectiveness.

Among the survey respondents, 94% answered that RMs have a positive effect on healthcare, and 76.5% answered that there is the possibility of a new treatment market based on the ability to conduct regenerative treatment. The respondents were aware of the growth potential of cell therapies and recognized their regenerative value. Regarding the optimal way to evaluate the market value of cell therapies, 64.7% of respondents answered that they recognized the results derived from interim results indicators, and 64.7% of respondents agreed with flexible application of the Institute for Clinical and Economic Review criteria. This shows that they recognized that the unique properties of cell therapies require a separate standard different from that of conventional drugs. However, opinions are divided regarding the choice to compare the price of cell therapies to that of comparable medicines when comparing costs; 47.1% of respondents said that cell therapies should be compared with the most expensive alternative drugs, while 41.2% of respondents said that cell therapies should be compared with the weighted average price of alternative drugs. Further discussions on comparative drug prices are expected to be needed in the future (Table 2).

**TABLE 2:** Recognition of RM’s value and how to decide pricing of RM.

Question	Respondents (n = 17)
Positive impact on healthcare	
Strongly agree	4 (23.5%)
Agree	12 (70.6%)
Disagree	1 (5.9%)
The Direction of RM affecting healthcare	
It can contribute to the development of healthcare	1 (5.9%)
There is a possibility for innovative new drugs with leading technologies	2 (11.8%)
RM has the potential to develop treatments in areas that have not been solved so far due to the possibility of regeneration. It will open up the possibility of a new treatment market.	13 (76.5%)
The need for value recognition of cell therapies	
Completely accept	5 (29.4%)
Partially accept	10 (58.8%)
No need for recognition	1 (5.9%)
Subjective scorecard	70.2 points
Screening criteria for value recognition of cell therapies: (Dual reply)	
Treatment for severe intractable diseases and incurable disease so far	15 (27.3%)
Domestic clinical trials	7 (12.7%)
Demonstrate clinical usefulness in phase 3 with clinically meaningful indicators	14 (25.5%)
Unique technology (e.g., patent)	3 (5.5%)
R&D investment ratio above average for innovative pharmaceutical companies	2 (3.6%)
Diseases that have no replaceable product and threaten patient’s survival	14 (25.5%)
Validating RM in accordance with global innovative new drug standards	
Appropriate	11 (64.7%)
Inappropriate	4 (23.5%)
Unknown	2 (11.8%)
Reason (a subjective question)	
Additional criteria needed for selecting a replacement drug for cell therapy	
Agree	13 (76.5%)
Disagree	2 (11.8%)
Unknown	1 (5.9%)
Adequacy of approval of results derived from interim results indicators	
Agree	11 (64.7%)
Disagree	5 (29.4%)
Strongly disagree	1 (5.9%)
Application of ICER for cell therapies	
Flexible application	11 (64.7%)
Existing critical with non-flexible application	5 (29.4%)
Unknown	1 (5.9%)
Number of comparative medicine’s prices when comparing RM costs	
The highest price of alternative drugs	8 (47.1%)
Weighted average price of alternative drugs	7 (41.2%)
Unknown	1 (5.9%)

## Discussion

Many international policies are aimed at accelerating the innovation of new drugs. We attempted to explore the gap between the market entry of RMs focused on cell therapy products and their reimbursement and clinical adoption.

Most regulators worldwide are realizing the need to accelerate approval plans to ensure early access to innovative treatments that can improve the quality of life for patients or perhaps even treat life-threatening conditions (Feigal et al., 2014). This is, in turn, reflected in regulatory legislation and policy. South Korea, Japan, the United States, and the European Union have enacted separate laws specifically for approval of RMs (Qiu et al., 2020).

However, for reimbursement of RM medicine, the uncertainty of evidence and their high upfront costs remains to be major challenges (Mahalatchimy, 2016). Above all, challenges posed by RMs include insufficient evidence, potential harm, lack of standardization in procedures, small target populations, and inadequate regulatory knowledge. Most RMs are specifically aimed at treating rare diseases, and small sample sizes of clinical trials unavoidably result in some uncertainty regarding the safety and efficacy of specific RMs. Second, approval data of RM are rarely obtained from single-arm studies. Furthermore, insufficient availability of evidence for evaluating the clinical effects of RMs prevents healthcare payers from negotiating reimbursement strategies with RM manufacturers (Abou-El-Enein et al., 2016). Third, health-related quality of life/utility data are lacking, which makes it impossible to accurately calculate quality-adjusted life years (QALYs) (Lloyd-Williams and Hughes, 2021). Lastly, while there is still a lack of evidence and policies to support the implementation of RMs, the pace of technological development is fast and investment is large (Bubela et al., 2015). As a result, there are only four cell therapies eligible for reimbursement among the 20 commercial products available in Korea. Failure to demonstrate cost-effectiveness has made it difficult to obtain reimbursement for cell therapies in most EU countries.

Therefore, our study confirmed that each stakeholder had a slightly different stance based on interviews and surveys about how to ensure patients’ access to RMs. To bridge the gap between approval and patient access, solutions for the failure of value-based assessments, such as those conducted by HTA bodies, need to be sought.

First, executives from pharmaceutical companies emphasized the need for price incentives due to increased manufacturing costs and the need for measures to consider their unmet needs, such as flexibility of the conventional reimbursement paradigm. The HIRA representatives and members of the Economic Evaluation Subcommittee were aware that it is necessary to evaluate RMs differently from the existing framework, since RMs could positively influence the pharmaceutical industry’s future investments. RMs have a positive effect on healthcare because there is a potential for them to meet unmet needs, in which the life science industry can develop new treatments in the form of regenerative drugs. However, innovative value is based on clinical usefulness, and RMs could not be evaluated under the traditional HTA framework at the current stage. Thus, an alternative HTA framework should be developed. Moreover, a national investment structure for RM treatments should be embedded into infrastructure rather than using preferential pricing or reimbursement.

Second, under a traditional reimbursement policy, the HIRA and PBCAC would review the market value of RMs using data on effectiveness or impact on QALYs. The final intended outcomes are typically used as a basis for the economic evaluation of new drugs (Bae et al., 2013). If economic modeling uses surrogate outcomes, such as readings in mmHg to indicate lower blood pressure, then indicators that are significantly associated with life extension should be widely used. In addition, health-related quality of life/utility data are lacking, thus making it impossible to calculate whether improvements in QALYs can be attributable to RMs.

Third, RMs require new value assessment, financing, and payment methods. Members of the PBCAC’s Economic Evaluation Subcommittee agreed to examine evidence of RMs effects using risk-based or outcome-based approaches. We concluded that the evidence requirements and decision-making considerations for evaluating RMs should align with the perspectives of regulatory bodies, health system payers, and developers. Previous studies mentioned that there is a considerable lack of clarity regarding which changes could successfully balance the competitive needs of industries, patients, regulators, and payers related to RMs (Bubela et al., 2015) and mechanisms for evaluating RMs based on real-world data or pay-for-performance models (Slocomb et al., 2017). RMs have mostly been approved after trials that included only a small number of patients (30–40 people). Since the level of evidence and effect size are low, most RMs are handled on a non-reimbursement basis. This seems to be due to difficulties such as high costs at the stage of clinical use by users such as doctors and patients and the process of producing patient-specific products rather than large-scale, standardized products. To stop the vicious cycle, conditional reimbursement to generate evidence of the effects of these medicines should be introduced. The conditional reimbursement method is a risk-sharing and refund system that returns the cost of the reimbursement received if the paid RM fails to generate evidence of its effectiveness.

Lastly, MFDS officials expressed concerns that RMs are unlikely to be subject to large-scale production standards and have high manufacturing costs, and that long-term enumeration results should be used for drugs subject to conditional approval. In the case of allogenic and xenogenic cell therapies, ongoing investment in these areas is necessary since they have the potential for mass production. However, since autologous cell therapies may require patient customization, it is considered difficult to mass-produce them, and it is necessary to examine them in future studies.

The significance of this study is that it collected opinions from executives and officials at pharmaceutical companies, regulatory bodies, and decision-making committees on the empirical difficulties related to reimbursement and pricing stages in South Korea’s health insurance system. Analyzing the current status of South Korea in terms of the difficulties faced in drug approval, marketing authorization, and reimbursement decision-making will have major implications for other countries. This study will serve as a foundation for drawing the best conclusions regarding how to protect patients and grow the healthcare market of cell therapies.

In addition, since we empirically studied the status of the actual use of RMs in the market, we believe that our findings have significant implications for other countries. Second, the difficulty of market valuation in the field of RM using an existing evaluation method has already been examined, mainly in the United Kingdom, Europe, and Japan (Hogarth and Salter, 2010; Ginty et al., 2011; Bubela et al., 2015; Abou-El-Enein et al., 2016; Faulkner, 2016; Lysaght and Sugii, 2016; Lysaght, 2017). This study also suggests a need for multidimensional consideration of the market value of RMs. Various alternatives, such as adaptation of economic assessment methods, conditional reimbursement, and implementing a risk-sharing system, were raised. Considering most of the experts interviewed from South Korea also agreed with the necessity of addressing unmet needs related to RMs, it is believed that further discussions on this topic will be necessary in the future.

Although there are few available RMs in the world currently, a large number of RMs are undergoing clinical trials, and the market for cell therapies is poised to grow rapidly very soon (Qiu et al., 2020). Many countries around the world are finding ways to expedite the approval of cell therapies. However, it is still relatively difficult to obtain reimbursement for RMs. There are limitations when it comes to the economic evaluation of RMs since there is a lack of evidence regarding the effectiveness of treatments. In countries where HTAs are being implemented, the cost-effectiveness of cell therapies must be proven for them to be eligible for reimbursement. Therefore, a method must be sought to overcome the gap between the market authorization and reimbursement processes. Infrastructure with appropriate reimbursement regimens and robust business models is not yet in place (Mason et al., 2011). There must be ways to introduce medical breakthroughs to the healthcare market while maintaining public health safety (Atkins et al., 2019).

In a broader context, the results of this study can be extended to the other countries in Asian region. In Eastern Asia Pacific and ASEAN region, economic growth continues, and various policies are being implemented to encourage numerous clinical trials of novel regenerative medicines are being in Japan, Korea, and China (McMahon et al., 2010; Sipp 2015; Cheng et al., 2016a; Takashima et al., 2021). Japan enacted Act on the Safety of Regenerative Medicine (ASRM) in 2014 (Fujita and Kawamoto 2016; Takashima et al., 2021), and has been working to construct a large-scale clinical registry (Okada et al., 2018). Japan allocated the 16.8 billion yen to RM research in 2017 fiscal year budget represents more than 10% of all biomedical research (Sipp, 2015). China established four stem cell banks, and State Food and Drug Administration (SFDA) announced the requirement for research of tissue-engineered medical products (Cheng et al., 2016a; Cheng et al., 2016b). Korea has also enacted a new law and tried to promote research. The results of this study are expected to be evidence as a benchmark for other countries in decision-making policy of reimbursement for RMs.

Although this study has several strengths, we were not able to recommend a way to change the existing reimbursement framework, which is a clear limitation. Also, patients and patient organizations are very much involved in the process of HTA and reimbursement decision-making in many jurisdictions. However, some patient groups raised their opinion about the need for reimbursement in the media; we did not included patient groups in qualitative study. Thus, further studies exploring this issue are needed.

## Data Availability

The raw data supporting the conclusion of this article will be made available by the authors, without undue reservation.
