# Supplementary material for: Regenerative Medicine in South Korea: Bridging the Gap Between Authorization and Reimbursement
Source: Front Bioeng Biotechnol. 2021 Aug 30;9:737504. doi: 10.3389/fbioe.2021.737504 (PMC8435711; doi:10.3389/fbioe.2021.737504)
Supplement: Supplementary file 1 [file Table1.docx]

Supplement. List of all market authorized RMs focused cell therapy products in South Korea

| No. | Product name | Origin | Target disease | Holder | ATC code | Approval date | Reimbursement date | Final Status |
| --- | --- | --- | --- | --- | --- | --- | --- | --- |
| 1 | Chondron | Chondrocyte | Knee cartilage defect | SEWON E & C Co., LTD | M09AX02 | 2001-01-30 | 2002-03-08 | Reimbursed |
| 2 | Holoderm | Skin keratinocyctes | Skin burn | TEGO & SCIENCE | D03 | 2002-12-10 | 2010-12-01 | Non-covered |
| 3 | Kaloderm (56㎠) | Skin keratinocyctes | Skin burn, diabetic foot ulcer | TEGO & SCIENCE | D03 | 2005-03-21 | 2005-03-21 | Reimbursed |
|  | Kaloderm (25㎠) |  |  |  |  |  | 2011-09-01 |  |
|  | Kaloderm (9㎠) |  |  |  |  |  | 2011-09-01 |  |
| 4 | Keraheal | Skin keratinocyctes | Skin burn | BIOSOLUTION | D03 | 2006-05-03 | 2010-12-01 | Non-covered |
| 5 | CreaVax-RCC Inj. | Autologous dendritic cells | Metastatic renal cell carcinoma | JW CREAZEN | L03AX | 2007-05-15 | 2010-12-01 | Non-covered |
| 6 | Immuncell-LC Inj. | Activating T lymphocytes | Liver cancer | GC CELL | L03AX | 2007-08-06 | 2011-01-01 | Non-covered |
| 7 | NKM Inj. | Activating lymphocytes | Malignant lymphoma | NK BIO | L03AX | 2007-08-07 | 2010-12-01 | Withdrawn |
| 8 | Hyalograft 3D | Autologous dermal fibroblasts | Diabetic foot ulcer | CHA MEDITEC | D03 | 2007-09-14 | 2010-12-01 | Withdrawn |
| 9 | RMS Ossron | Bone cell | Promotion of local bone formulation | SEWON E & C Co., LTD | M05BX | 2009-08-26 | 2010-12-01 | Non-covered |
| 10 | Autostem | Autologous adipose tissue-derived minimally engineered fat cells | Subcutaneous fat defect | CHA MEDITEC | D03 | 2010-02-01 | 2010-12-01 | Withdrawn |
| 11 | Quenncell | Fat cell | Subcutaneous fat defect | ANTROGEN | D03 | 2010-03-26 | 2010-12-01 | Non-covered |
| 12 | Cure skin | Fibroblast | Relief of scars involved in acne healing | S.BIOMEDICS | D03 | 2010-05-11 | 2011-02-01 | Non-covered |
| 13 | LSK Autograft | Autologous skin keratin cells | Skin burn | S.BIOMEDICS | D03 | 2010-09-17 | 2011-03-01 | Withdrawn |
| 14 | Hearticellgram-AMI | Autologous bone marrow derived mesenchymal stem cells | Improving left ventricular blood rate in myocardinal infarction | PHARMICELL | C01 | 2011-07-01 | 2016-06-01 | Non-covered |
| 15 | Cartistem | Umbilical cord blood derived mesenchymal stem cells | Treatment of knee carilage defects in osteoarthritis due to degeneration | MEDI-POST | B05AX04 | 2012-01-18 | 2012-05-01 | Non-covered |
| 16 | Cupistem | Fat-derived mesenchymal stem cells | Fistula treatment due to Crohn's disease | ANTROGEN | A16A | 2012-01-18 | 2016-01-01 | Reimbursed |
| 17 | Neuronata-R inj. | Bone marrow derived mesenchymal stem cells | Alleviation of disease progression in patients with amyotrophic lateral sclerosis | CORESTEM | B05AX | 2014-07-30 | 2014-10-01 | Non-covered |
| 18 | Keraheal-Allo | Skin keratinocytes | Promotion of re-epithelialization of deep second degree burns | BIOSOLUTION | D03 | 2015-10-16 | 2016-01-01 | Reimbursed |
| 19 | Rosmir | Skin-derived fibroblasts | Improvement of more than severe nasojugal groove | TEGO & SCIENCE | D03 | 2017-12-27 | 2018-11-01 | Non-covered |
| 20 | CartiLife | Chondrocyte | Knee cartilage defect | BIOSOLUTION | M09AX02 | 2019-04-24 | 2021-07-01 | Non-covered |
