# Supplementary figures and images for: Regenerative Medicine in South Korea: Bridging the Gap Between Authorization and Reimbursement
Source: Front Bioeng Biotechnol. 2021 Aug 30;9:737504. doi: 10.3389/fbioe.2021.737504 (PMC8435711; doi:10.3389/fbioe.2021.737504)

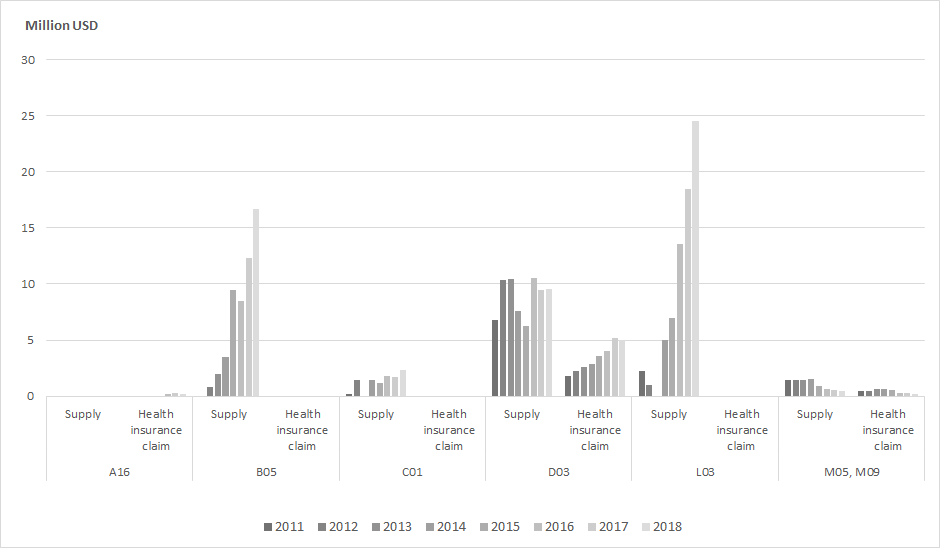

Supplement: Supplementary file 2 [file Figure2.TIF]
